# Dohaekseunggi-tang extract inhibits obesity, hyperlipidemia, and hypertension in high-fat diet-induced obese mice

**DOI:** 10.1186/1472-6882-14-372

**Published:** 2014-10-04

**Authors:** Yoon-Young Sung, Dong-Seon Kim, Goya Choi, Seung-Hyung Kim, Ho Kyoung Kim

**Affiliations:** Herbal Medicine Resources Group, Herbal Medicine Research Division, Korea Institute of Oriental Medicine, 1672 Yuseong-daero, Yuseong-gu, Daejeon, 305-811 Republic of Korea; Institute of Traditional Medicine and Bioscience, Daejeon University, Daejeon, 300-716 Republic of Korea

**Keywords:** Angiotensin-1 converting enzyme, Body weight, Dohaekseunggi-tang, High-fat diet, Pancreatic lipase, Visceral adipose tissue

## Abstract

**Background:**

Dohaekseunggi-tang (DHSGT) is a traditional plant-based medicine prescribed to promote blood circulation and to treat obesity and hypertension. The present study aimed to identify potential anti-obesity activities of DHSGT extract.

**Methods:**

Anti-obesity, anti-hyperlipidemic, and anti-hypertensive effects of orally-administered DHSGT extract were evaluated in high-fat diet- (HFD)-induced obese mice. Serum biochemistry profiles and expression of diverse metabolic regulatory gene mRNAs in mouse visceral fat were assessed by RT-PCR. The effects of DHSGT on angiotensin-1 converting enzyme (ACE) and pancreatic lipase activities were determined using *in vitro* inhibition assays.

**Results:**

Oral DHSGT treatment reduced obese HFD C57BL/6 J mouse body weight, liver and adipose tissue mass, adipocyte size, and blood pressure versus untreated HFD mice. DHSGT also decreased serum total cholesterol, LDL-cholesterol, triglyceride, glucose, and leptin concentrations, and increased HDL-cholesterol and adiponectin levels in HFD mice. Furthermore, DHSGT markedly increased mRNA expression of peroxisome proliferator activated receptor-gamma, uncoupling protein-2, and adiponectin in visceral adipose tissue of HFD mice. *In vitro* tests revealed that DHSGT effectively inhibited porcine pancreatic lipase and ACE activities, with IC_50_ values of 7.58 mg/ml and 0.56 mg/ml, respectively.

**Conclusions:**

These results validate traditional knowledge and suggest that DHSGT may be potentially useful for managing hyperlipidemia, hyperglycemia, hypertension, and obesity.

## Background

Obesity is associated with an imbalance between energy intake and energy expenditure and a subsequent accumulation of excess fat [1]. Obesity is a major risk factor for metabolic diseases such as hyperlipidemia, hypertension, atherosclerosis, type 2 diabetes, and fatty liver [2]. Pharmacological agents such as appetite-suppressants, fat absorption suppressants, and thermogenic drug are often prescribed for weight loss in treating obesity [3]. Orlistat, which reduces dietary fat absorption by inhibiting pancreatic lipase, is currently on the global market [4]. Pancreatic lipase is a major enzyme involved in triglyceride absorption in the intestine, and inhibiting fat absorption from the diet is a target for treating obesity [5]. However, these drugs have many limitations due to adverse effects, such as stomach pain, steatorrhea, and headaches [6]. Recently, the high prevalence of obesity and associated complications such as hypertension has led to increased research in traditional herbal medicines that have negligible adverse effects, as alternative weight-loss therapies [7].

In symptom-based Oriental medicine, the underlying treatment mechanism of obesity is accomplished as follows: remove dampness, dispel phlegm, induce diuresis, remove internal heat, promote digestion and remove food stagnancy, restore the normal flow of the liver Qi (vital energy), and invigorate the spleen [8, 9]. Dohaekseunggi-tang (DHSGT) (Taohe Chengqi Tang: Chinese), is a traditional medicine prescribed for oral administration to drain heat and promote blood flow to eliminate blood stasis [10]. In Korea, it is mainly prescribed to treat obesity and hypertension caused by stagnation of phlegm-dampness and blood stasis [3, 11]. DHSGT has also been documented to treat diabetes mellitus, chronic pyelonephritis, chronic hepatitis, acute necrotic enteritis, and amenorrhea [12] .This prescription is an herbal mixture composed of *Glycyrrhizae uralensis* Fischer (Chinese licorice), *Rheum undulatum* Linne (a rhubarb relative), *Prunus persica* L. (peach tree), and *Cinnamomum cassia* Presl (Chinese cinnamon, an evergreen tree) [13]. Recently, DHSGT was reported to ameliorate diabetic atherosclerosis in western diet-fed apolipoprotein E knockout (ApoE KO) mice [14]. Treatment with DHSGT in western diet-fed ApoE KO mice also reduced LDL cholesterol, triglyceride, and glucose levels as well as blood pressure [14]. In high-fat diet-induced obese rats, DHSGT decreased the body weight, liver and adipose tissue weights, and serum triglyceride levels [15]. DHSGT also decreased plasma glucose, cholesterol, triglyceride, and LDL cholesterol levels in high-fat/high-cholesterol diet-fed *db/db* mice [16]. These results show that DHSGT may be useful in the treatment of obesity and metabolic disease. However, DHSGT effects and mechanisms of action in treating obesity remain obscure. Here, we investigated the potential anti-obesity and anti-hypertensive effects of DHSGT using a high-fat diet-induced mouse model and *in vitro* assays to evaluate possible DHSGT inhibition of pancreatic lipase and angiotensin-1-converting enzyme (ACE) activities.

## Methods

### Preparation of DHSGT

The herbs were purchased from Kwangmyeongdang Medicinal Herbs Co. (Ulsan, Korea) and authenticated based on their microscopic and macroscopic characteristics by the Classification and Identification Committee of the Korea Institute of Oriental Medicine (KIOM). The formula for DHSGT consists of five herbs including *Glycyrrhizae uralensis* Fischer (40 g), *Rheum undulatum* Linne (80 g), *Prunus persica* Linne (60 g), *Cinnamomum cassia* Presl (40 g), and Natrii Sulfas (40 g) mixed. The DHSGT (260 g) was boiled with distilled water (1:10, v/v) at 100°C for 2 h, and the extract was filtered, lyophilized, and subsequently stored at -20°C. The yield of DHSGT aqueous extract was 11.25% (w/w).

### Animals and experimental diets

Male, 8-week-old C57BL/6 J mice were purchased from The Jackson Laboratory (Bar Harbor, ME, USA). They were housed in an air-conditioned room with a 12-h light/12-h dark cycle at a temperature of 21 ± 2°C and humidity of 50 ± 5%, and were fed a commercial diet (Ralston-Purina, St. Louis, MO, USA) for 1 week. Mice were provided with a laboratory diet and water ad libitum. For inducing obesity, the mice were fed a high-fat diet (Rodent Diet D12492; Research Diets, Inc., New Brunswick, NJ, USA), providing 60% of energy as fat, 20% as protein, and 20% as carbohydrates. Non-obese control mice were fed a commercial standard chow diet (Orient Bio Inc., Seongnam, Korea) consisting of 14% energy as fat, 21% as protein, and 65% as carbohydrates. The mice were randomly divided into three groups (n = 5), and respectively fed a normal diet (Control), a high-fat diet (HFD) and a high-fat diet plus DHSGT (HFD-DHSGT) for 7 weeks. DHSGT was dissolved in normal saline, and was orally administered to the mice (200 mg/kg/day). The Control and HFD mice received vehicle (normal saline) only. Body weight and food intake were monitored every week. This study adhered to the Guide for the Care and Use of Laboratory Animals developed by the Institute of Laboratory Animal Resources of the National Research Council, and was approved by the Institutional Animal Care and Use Committee of Daejeon University in Daejeon, Korea.

### Biochemical serum assay

At the end of the experiment period, the mice were anesthetized with ether after an overnight fast. Blood was withdrawn from the abdominal aorta into a vacuum tube. Biochemical analyses of concentrations of total cholesterol, HDL cholesterol, LDL cholesterol, triglyceride, glucose, creatinine, aspartate aminotransferase (AST), and alanine aminotransferase (ALT) were determined using an automatic analyzer (Express Plus; Chiron Diagnostics, East Walpole, MA, USA). Serum insulin, leptin, and adiponectin levels were measured by immunoassay (ELISA) using commercially available kits (Linco Research Inc., St. Charles, MO, USA).

### Weight and histology analysis of adipose tissues and liver

After blood collection, the white adipose tissues (subcutaneous and visceral fat) and liver were removed from each mouse and weighed. For adipocyte staining, visceral adipose tissue was fixed in 10% neutral-buffered formalin solution for 1 day, paraffin-embedded, sectioned to a thickness of 6 μm, and stained with hematoxylin and eosin. To quantitate adipocyte size, the stained sections were analyzed using light microscopy (Olympus BX51, Olympus Optical Co., Tokyo, Japan) and image analysis software (Image-Pro Plus version 5.0; Media Cybernetics, Silver Spring, MD, USA).

### Real-time quantitative RT-PCR

Total RNA from visceral adipose tissue was isolated with TRI reagent® (Sigma-Aldrich, St. Louis, MO, USA) and digested with DNase I (Life Technologies, Grand Island, NY, USA) to remove chromosomal DNA. Five micrograms of total RNA was reverse transcribed into cDNA with the First Strand cDNA Synthesis kit (Amersham Pharmacia, Piscataway, NJ, USA). Real-time quantitative PCR was performed using the Applied Biosystems 7500 Real-Time PCR system (Applied Biosystems, Grand Island, NY, USA). The primer sequences and the probe-sequence are shown in Table 1. Probes were labeled with the fluorescent reporter 6-carboxy-fluorescein. Aliquots of sample cDNAs and an equal amount of beta-actin cDNA were amplified with the TaqMan® Universal PCR master mixture containing DNA polymerase according to manufacturer instructions (Applied Biosystems, Foster, CA, USA). PCR conditions were 2 min at 50°C, 10 min at 95°C, 15 s at 95°C, and 1 min at 60°C for 40 cycles. The concentration of target gene was determined using the comparative Ct (threshold cycle number at cross-point between amplification plot and threshold) method, according to manufacturer instructions.

**Table 1:** **Sequences of probe and oligonucleotides used in real-time RT-PCR analysis**

Genes	Probe and Primer	Sequence
Leptin	sense	5′-AACCCTTACTGAACTCAGATTGTTAG-3′
	antisense	5′-TAAGTCAGTTTAAATGCTTAGGG-3′
UCP-2	sense	5′-TTCAAATGAGATTGTGGGAAAAT-3′
	antisense	5′-ACCGATACAGTACAGTACAGTA-3′
Adiponectin	sense	5′-CCCAAGGGAACTTGTGCAGGTTGGATG-3′
	antisense	5′-GTTGGTATCATGGTAGAGAAGAAAGCC-3′
PPAR-γ	FAM	5′-TCGGAATCAGCTCTGTGGACCTCTCC-3′
GAPDH	VIC	5′-TGCATCCTGCACCACCAACTGCTTAG-3′

### Blood pressure measurement

Blood pressure was determined by a tail-cuff method using the CODA™ noninvasive blood pressure cuff system (Kent Scientific Corp., Torrington, CT, USA).

### Pancreatic lipase activity inhibition assay

The ability of DHSGT to inhibit pancreatic lipase activity was measured using the QuantiChrom™ lipase assay kit (BioAssay Systems, Hayward, CA, USA). The colorimetric enzyme assay is based on the dimercaptopropanol tributyrate (BALB) and 5,5′-dithiobis 2-nitrobenzoic acid (DTNB) method of Furukawa et al. [17]. DHSGT was dissolved in water and serially diluted. After incubating 10 ul of DHSGT sample solution at different concentrations (0, 0.5, 1, 2, 6.25, 12.5 or 25 mg/ml) or water (control, no inhibition) and 5 ul of porcine pancreatic lipase solution (50 units/ml) with 140 ul of working solution for 10 min and 20 min at room temperature, sample absorbance was measured at 412 nm using a microplate reader (Multiskan® GO; Thermo Fisher Scientific, Hudson, NH, USA). The working solution was prepared by the addition of 5 mg color DTNB reagent and 8 ul substrate BALB solution (20 mM BALB in ethanol) in 140 ul Tris buffer (100 mM Tris–HCl and 5 mM CaCl_2_, pH 8.5). Lipase activity was determined by measuring the SH group formed from lipase cleavage of BALB react with DTNB to yellow colored product. Orlistat (0, 0.01, 0.1, 0.5 or 1 mg/ml) was used as the positive control. Lipase inhibition (%) was calculated according the following formula: Inhibition activity (%) = {[OD (20 min-10 min)_control_-OD (20 min-10 min)_sample_] / OD (20 min-10 min)_control_} × 100. The IC_50_ value was defined as the concentration of inhibitor required to inhibit 50% of pancreatic lipase activity.

### ACE activity inhibition assay

ACE activity was measured using the K-assay® ACE inhibition kit (Kamiya Biomedical Co., Seattle, WA, USA). DHSGT was dissolved in water and serially diluted. Twenty μl of DHSGT sample solution at different concentrations (0, 0.03, 0.06, 0.125, 0.25, 0.5, 1 or 2 mg/ml) or water (control, no inhibition) were added to 20 μl of 3HB-GGG substrate buffer (0.69 mM 3-hydroxybutyryl-Gly-Gly-Gly in 50 mM HEPES, pH 8.3). Twenty μl of ACE enzyme working solution (25 units/ml) was then added and the mixture was incubated at 37°C for 60 min. Then, 200 μl of indicator WST (a water soluble tetrazolium dye) solution was added to the reaction mixture and incubated at room temperature for another 10 min. The absorbance at 450 nm was measured the amount of cleaved 3-hydroxybutyric acid (3HB) from 3HB-GGG using a microplate reader. Captopril at same concentrations was used as positive control. ACE inhibition (%) was calculated according the following formula: Inhibition activity (%) = [(OD_control_-OD_sample_) / OD_control_] × 100. The IC_50_ value was defined as the concentration of inhibitor required to inhibit 50% of ACE activity.

### Statistical analysis

Data were analyzed by one-way analysis of variance followed by Duncan’s multiple-range tests. All data are presented as means ± SE. Significant differences were accepted when the p-value was <0.05.

## Results

### Changes of body weight, food intake and food efficiency ratio

**Figure 1:**
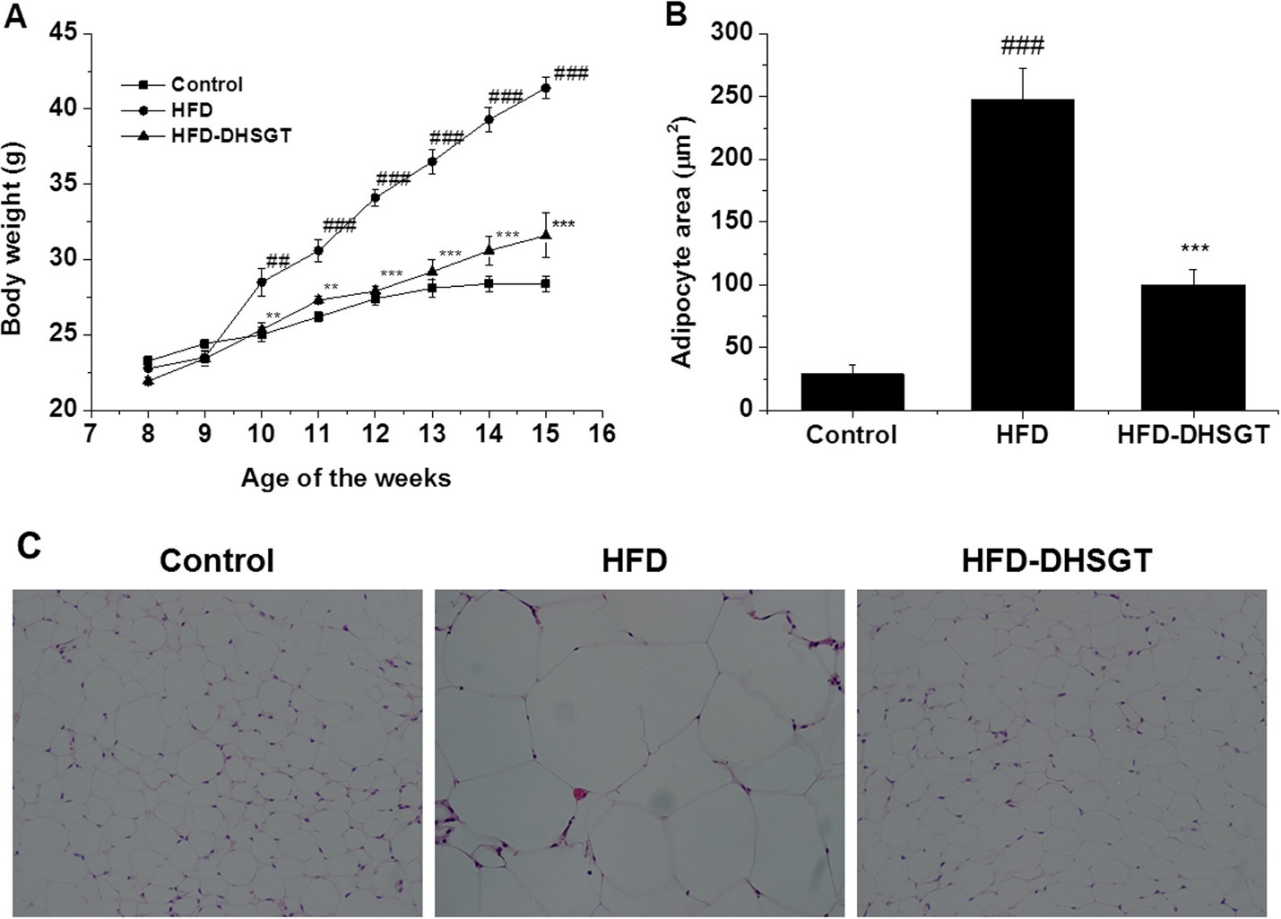
**Effect of DHSGT on body weight and adipocyte size in HFD-induced obese mice. (A)** Body weight in obese mice fed HFD for 7 weeks. **(B)** Adipocyte area, and **(C)** representative images of hematoxylin and eosin-stained visceral fat sections (original magnification 400×). Values are expressed as means ± SEM (n = 5 animals/group). Significant differences were observed between the Control and HFD groups: ##, *p* < 0.01; ###, *p* < 0.001. Significant differences were observed between the HFD and HFD-DHSGT groups: **, *p* < 0.01; ***, *p* < 0.001. HFD, high-fat diet; DHSGT, Dohaekseunggi-tang.

Body weight, body weight gain, food intake, and food efficiency ratio of the mice fed the HFD with oral treatment of DHSGT for 7 weeks shown in Figure 1A and Table 2. The final body weight of mice fed the HFD was higher than that of mice fed the normal diet and the DHSGT treatment reduced body weight compared to the high- fat diet group. Body weight gain was significantly decreased in the DHSGT treated group compared to the HFD control group. DHSGT did significantly not affect food intake. Food efficiency ratio (FER) was higher in the HFD group than in the Control group, and the FER was significantly decreased in the DHSGT-treated group versus the HFD group. These results indicate that the DHSGT treatment efficiently reduces body weight increases caused by the high-fat diet.

**Table 2:** **Effects of DHSGT on body weight, food intake, food-efficiency ratio, and tissue weight**

	Control	HFD	HFD-DHSGT
Final body weight, g	28.40 ± 0.51	41.40 ± 0.68###	31.60 ± 1.47***
Body-weight gain, g/day	0.09 ± 0.01	0.37 ± 0.02###	0.17 ± 0.04***
Food intake, g/day	3.36 ± 0.00	2.69 ± 0.00	2.24 ± 0.00
Food-efficiency ratio (%)	2.64 ± 0.31	13.60 ± 0.57###	7.53 ± 1.58**
White adipose-tissue weight			
Subcutaneous, g	0.29 ± 0.08	1.92 ± 0.12###	0.93 ± 0.45**
Visceral, g	0.28 ± 0.03	3.79 ± 0.27###	1.46 ± 0.30***
Liver weight, g	1.04 ± 0.07	1.12 ± 0.04	0.85 ± 0.06**

Food-efficiency ratio (%) = (body-weight gain/food intake) × 100. Values are expressed as means ± SEM (n = 5); ###, *p* < 0.001 compared with the Control group; **p < 0.01; ***, *p* < 0.001 compared with the HFD group.

### Weights of adipose tissue and liver

To investigate whether DHSGT decreases adiposity, fat tissues and liver were removed from mice and weighed. Subcutaneous and visceral fat weights were increased in the HFD mice by 6.6 and 13.5-fold, respectively, versus Control mice, and this effect was significantly mitigated by treating HFD mice with DHSGT (Table 2). Liver weight was decreased in the HFD-DHSGT treated mice versus HFD mice (Table 2).

### Biochemical determinations in serum

Serum biochemical profiles of all three mouse groups after receiving their respective diets for 7 weeks are shown in Table 3. The triglyceride, total cholesterol, and LDL-cholesterol levels of mice fed the high-fat diet were higher than those of mice fed the normal diet. DHSGT treatment significantly suppressed serum lipid concentration increases in HFD mice. Glucose levels in HFD mice were higher than those of mice fed the normal diet, and DHSGT treatment significantly suppressed HFD-induced serum glucose elevation, to levels approximating those in untreated Control mice. However, HDL-cholesterol concentrations were not significantly different between the HFD and HFD-DHSGT groups.

**Table 3:** **Biochemical measurements in mouse serum**

	Control	HFD	HFD-DHSGT
Total cholesterol, mg/dl	98.6 ± 3.7	163.2 ± 13.3##	130.2 ± 4.4*
Triglyceride, mg/dl	27.0 ± 1.3	33.4 ± 1.8#	27.4 ± 1.5*
LDL cholesterol, mg/dl	6.4 ± 0.7	13.8 ± 1.4##	7.0 ± 0.4**
HDL cholesterol, mg/dl	63.0 ± 0.8	75.0 ± 2.7##	69.8 ± 2.3
Glucose, mg/dl	134.8 ± 3.00	200.2 ± 11.5##	144.4 ± 19.3*
AST, U/l	80.8 ± 4.3	112.6 ± 10.4#	86.8 ± 5.3
ALT, U/l	39.6 ± 2.4	58.2 ± 3.7 ##	32.0 ± 3.9**
Creatinine, mg/dl	0.142 ± 0.015	0.172 ± 0.009	0.140 ± 0.008*
Leptin, ng/ml	0.14 ± 0.02	7.35 ± 0.46 ###	1.50 ± 1.12***
Adiponectin, ng/ml	1.39 ± 0.24	3.61 ± 0.29###	13.46 ± 2.43***

Values are expressed as means ± SEM (n = 5). #, *p* < 0.05; ##, *p* < 0.01; ###, *p* < 0.001 compared with the Control group; *, *p* < 0.05; **, *p* < 0.01; **, *p* < 0.01 compared with the HFD group.

To evaluate potential toxic effects of ingesting DHSGT, serum markers that indicate liver and kidney damage were measured at the end of the experimental period (Table 3). Creatinine levels were significantly decreased in the HFD-DHSGT mice versus levels in HFD mice. Serum levels of AST and ALT were increased in HFD mice compared to Control mice. Levels of ALT were significantly decreased in the HFD mice by DHSGT-treatment mice but AST levels were not significantly affected by DHSGT-treatment in HFD mice.

### Adipocytokine serum concentrations

Mice fed the HFD displayed approximately 50-fold increased serum leptin levels versus Control mice (Table 3). Treatment of HFD mice with DHSGT decreased HFD-induced serum leptin by approximately 80%. Serum adiponectin was increased less than 2-fold in HFD mice versus normal-diet Control mice. Treatment of HFD mice with DHSGT caused an additional increase in serum adiponectin, to levels nearly four-fold higher than levels measured in HFD-only mice (Table 3).

### Histology of adipose tissue

Average visceral fat adipocyte size (area) in HFD mice was significantly larger (approximately10-fold) than in normal-diet Control mice (Figure 1B and C). When HFD mice were treated with DHSGT, adipocyte size was significantly decreased by over 50%. These results indicate that DHSGT-induced reduction in fat mass was partly due to decreased visceral fat adipocyte size.

### Expression of genes involved in lipid metabolism

**Figure 2:**
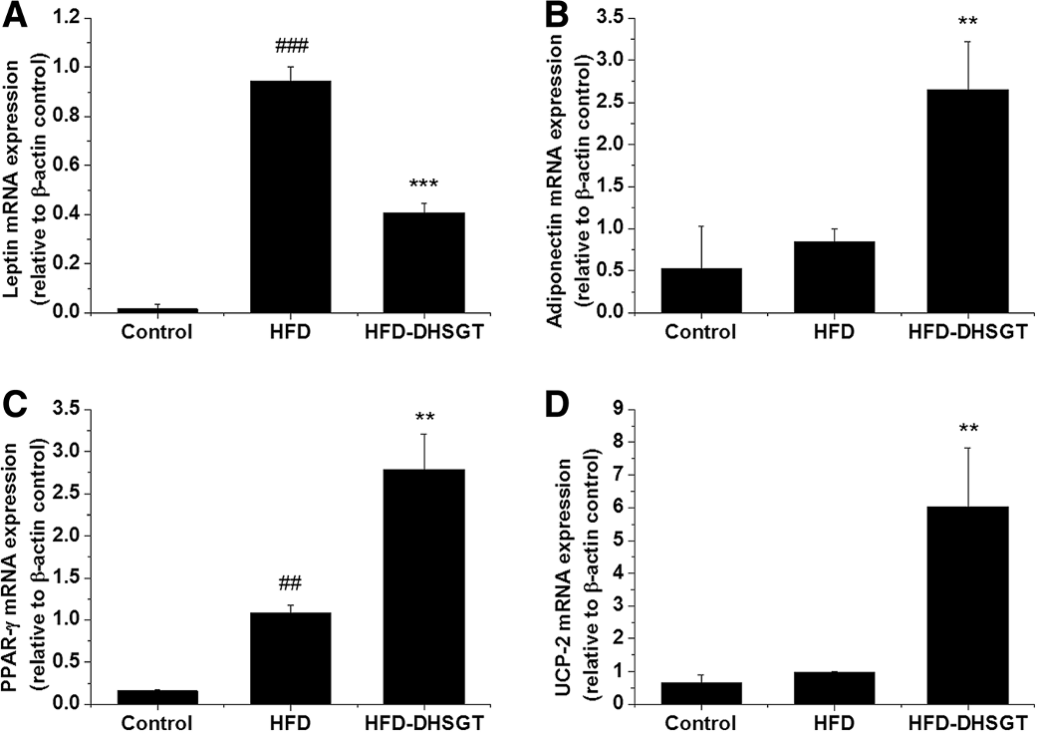
**Effect of DHSGT on mRNA expression of leptin, adiponectin, PPAR-γ, and UCP-2 in visceral adipose tissue of HFD-induced obese mice.** Expression levels of **(A)** leptin, **(B)** adiponectin, **(C)** PPAR-*g*, and **(D)** UCP-2. Values are expressed as means ± SEM (n = 5 animals/group). Significant differences were observed between the Control and HFD groups: ##, *p* < 0.01; ###, *p* < 0.001. Significant differences were observed between the HFD and HFD-DHSGT groups, respectively: **, *p* < 0.01; ***, *p* < 0.001. DHSGT, Dohaekseunggi-tang; PPAR-γ, peroxisome proliferator activator receptor-γ; UCP-2, mitochondrial uncoupling protein-2, HFD, high-fat diet.

To understand the mechanism of DHSGT effects on lipid metabolism, we investigated expression of lipogenesis- or lipolysis-related genes in visceral fat (Figure 2). The HFD increased leptin mRNA expression versus Control mice and DHSGT-treatment decreased HFD-induced leptin mRNA by >50% (Figure 2A). While HFD mice expressed similar adiponectin mRNA levels versus Control mice, DHSGT-treatment of HFD mice significantly increased adiponectin (Figure 2B). Peroxisome proliferator activated receptor-γ (PPAR-γ) mRNA expression was significantly increased in HFD mice versus Control mice, and was further increased by DHSGT-treatment of HFD mice (Figure 2C). Expression of mitochondrial uncoupling protein-2 (UCP-2) mRNA, a molecule involved in fat metabolism, was not affected by HFD alone, but was markedly upregulated in HFD mice that were concomitantly treated with DHSGT (Figure 2D).

### Pancreatic lipase inhibition

**Figure 3:**
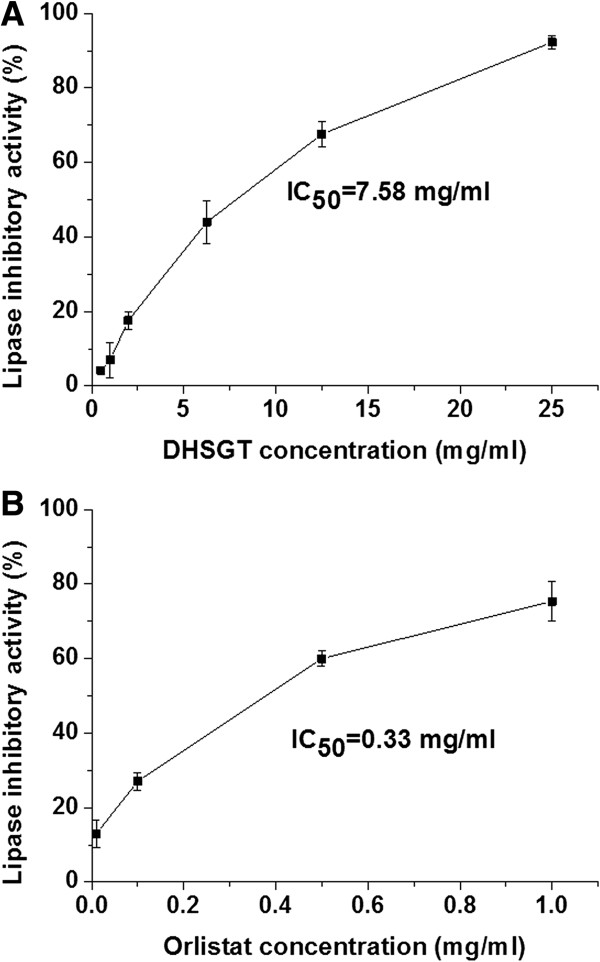
**Inhibitory effect of DHSGT on** ***in vitro*** **porcine pancreatic lipase activity. (A)** Pancreatic lipase inhibitory activity at different concentration of DHSGT. **(B)** Orlistat was used as a positive control. Values are expressed as means ± SEM of three independent experiments. DHSGT, Dohaekseunggi-tang.

The DHSGT extract exhibited pancreatic lipase inhibitory activity in a concentration-dependent manner (Figure 3A). DHSGT at 25 mg/ml inhibited pancreatic lipase activity by 92.28%, while orlistat (1 mg/ml, positive control) inhibited 75.38% of the enzyme activity (Figure 3B). The IC_50_ values of DHSGT extract and orlistat were 7.58 mg/ml and 0.33 mg/ml, respectively.

### ACE inhibition

**Figure 4:**
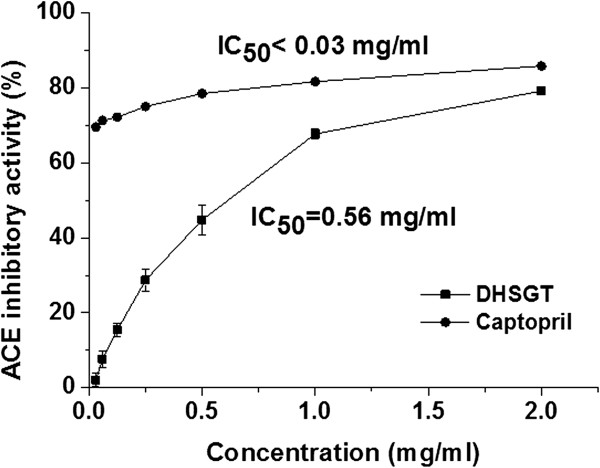
**Inhibitory effect of DHSGT on angiotensin 1-converting enzyme** ***in vitro*** **.** Captopril was used as a positive control. Values are expressed as means ± SEM of three independent experiments. DHSGT, Dohaekseunggi-tang.

ACE activity was inhibited by DHSGT extract in a concentration-dependent manner (Figure 4). Two mg/ml DHSGT caused 77.06% inhibition, while 2 mg/ml captopril (positive control) caused 85.78% inhibition. The ACE inhibition IC_50_ values of DHSGT extract was 0.56 mg/ml, while captopril had an IC_50_ value <0.03 mg/ml (Figure 4).

### Effect of DHSGT on blood pressure

Systolic, diastolic, and mean blood pressure measurements were all markedly upregulated in HFD mice versus normal-diet control mice (Table 4). All three parameters were significantly decreased in HFD mice that concurrently received DHSGT treatment.

**Table 4:** **Effects of DHSGT on blood pressure**

	Control	HFD	HFD-DHSGT
Blood pressure			
Systolic, mmHg	65.36 ± 6.30	170.56 ± 06.33###	86.56 ± 7.39***
Diastolic, mmHg	61.14 ± 4.97	167.00 ± 05.80###	82.33 ± 7.26***
Mean, mmHg	62.43 ± 5.25	101.11 ± 13.57###	83.67 ± 7.22*

Values are expressed as means ± SEM (n = 5 animals per group). ###, *p* < 0.001 compared with the Control group; *, *p* < 0.05; ***, *p* < 0.001 compared with the HFD group.

## Discussion

Adipose tissue is a dynamic organ that plays an important role in energy balance and body mass changes throughout an individual’s lifetime, in response to the metabolic requirements of the organism [18]. This study investigated the effects of DHSGT on HFD-induced fat accumulation in the adipose tissue of mice. In mice made obese by feeding a high-fat diet, DHSGT treatment inhibited white adipose tissue accumulation and adipocyte enlargement, and, decreased body-weight gain and serum total cholesterol, LDL-cholesterol, triglyceride, and glucose levels.

Adipocytes secrete various adipocytokines such as tumor necrosis factor-α, leptin, adiponectin, and resistin [19]. Adiponectin plays important roles in insulin sensitivity and fatty acid oxidation, and adiponectin levels are negatively correlated with body fat mass, and serum glucose, insulin, and triglyceride levels [20]. Leptin levels in human and rodent are directly associated with adiposity and body-weight changes [21]. In this study, both the serum adiponectin protein levels and adipocyte adiponectin mRNA levels were markedly increased in DHSGT-treated obese mice, whereas leptin expression in serum and adipose tissues was decreased by DHSGT treatment. These results suggest that improved adipocytokines levels by DHSGT treatment may be contribute to decreased body weight, body fat accumulation, and serum levels of glucose, cholesterol, and triglycerides. Obesity due to adipocyte hypertrophy leads to changes in adipocytokine profiles involved in the development of insulin resistance, as well as in the production of signaling molecules, such as PPAR-γ, aP2, and leptin [22]. PPAR-γ is a transcription factor prominently expressed in adipose tissue, and it activates adipocyte differentiation [23]. Moreover, exposure of mice to high fat diet increases adipose tissue expression of PPAR-γ [24]. Our result also showed that the HFD-induced obese mice expressed higher mRNA levels of PPAR-γ then the normal control mice: however, these effects were weaker than that of the DHSGT-treated mice. In this study, feeding the HFD produced slight increases in serum adiponectin levels. Circulating adiponectin levels were known to be modulated by PPAR-γ [25]. Thus, the increases of adiponectin levels in serum of HFD mice may be mediated by the up-regulation of PPAR-γ. Treatment with DHSGT increased mRNA expression of PPAR-γ and UCP-2 in visceral adipose tissue. Thiazolidinediones (TZDs) improve insulin sensitivity through PPAR-γ, and TZD treatment in obese humans increases adiponectin levels [26]. In rodent disease models, PPARγ agonists prevent increased adiposity and body weight, and improve insulin resistance and dyslipidemia [27]. DHSGT treatment restored decreased PPAR-γ expression levels in the aorta of western diet-fed ApoE KO mice and the beneficial effect of DHSGT on endothelial dysfunction in diabetic atherosclerosis was similar to rosiglitazone (a PPARγ agonist) which is ameliorated in insulin sensitivity [14]. UCP-2 plays an important role in fat metabolism by promoting fatty acid oxidation in white adipose tissue and reducing body-weight gain [28]. Decreased fat accumulation and serum lipid levels by DHSGT treatment are possibly mediated by increased expression of these regulatory genes.

Pancreatic lipase is a key enzyme that hydrolyzes 50-70% of total dietary fat in the digestive system, converting triglycerides to monoglycerides and free fatty acids [7, 29]. Inhibiting pancreatic lipase is an important strategy for treating obesity and other metabolic disorders. In this study, DHSGT inhibited pancreatic lipase activity in a concentration-dependent manner, with an IC_50_ value of 7.58 mg/ml. The lipase inhibitory activity of DHSGT may be able to suppress dietary fat absorption *in vivo*, too.

Obesity is a worldwide disease that is often accompanied by several metabolic abnormalities such as hypertension, hyperglycemia, and dyslipidemia [30]. The renin-angiotensin-aldosterone system (RAAS) is a major mediator of hypertension. The RAAS system produces angiotensin II from angiotensin I, and angiotensinogen via renin and ACE [31]. Additionally, ACE metabolizes and inactivates the vasodilator bradykinin. Thus, ACE has dual roles of increasing vasoconstriction and inactivating vasodilation [32]. DHSGT showed potent inhibitory activity against ACE *in vitro*, with an IC_50_ value of 0.56 mg/ml, and it decreased the high blood pressure induced by increasing dietary fat in the HFD mouse group. This indicates a potential anti-hypertensive effect of DHSGT. Activation of the RAAS is common in obesity [33]. Increased adipose tissue RAAS activity by diet-induced obesity promotes inflammation, lipogenesis and reactive oxygen species generation, and impairs insulin signaling, all of which worsen the adipose environment [34]. Thus, RAAS pathway blockade by inhibiting ACE activity is one goal in treating the hypertension characteristic of metabolic syndrome and obesity. This study evaluated the ACE inhibitory effects of DHSGT extract using an *in vitro* model. For natural products to inhibit ACE, they first need to be absorbed in the blood and then act ACE. Since we are evaluating a blend of herbal extracts that contains numerous bioactive compounds, further study is needed to identify and evaluate the active ACE inhibitory compounds in the DHSGT extract.

## Conclusions

DHSGT treatment ameliorates body-weight gain, adipose tissue accumulation, hypertension, and dysregulated serum lipid profiles in HFD-induced obese mice by improving lipid metabolism. Our *in vitro* assay showed that DHSGT is an effective inhibitor of pancreatic lipase and ACE activity. These findings validate traditional knowledge and suggest that DHSGT may potentially be useful for managing hyperlipidemia, hyperglycemia, hypertension, and obesity. Thus, further study will be performed to identify and characterize the bioactive components responsible for its anti-obesity and anti-hypertensive effects.

## Abbreviations

ACE: Angiotensin-1 converting enzyme
ALT: Alanine aminotransferase
AST: Aspartate aminotransferase
DHSGT: Dohaekseunggi-tang
HDL: High density lipoprotein
LDL: Low density lipoprotein
PPARγ: Peroxisome proliferator-activated receptor γ
RAAS: Renin-angiotensin-aldosterone system
UCP: Uncoupling protein.
